# Controllability, Matching Ratio and Graph Convergence

**DOI:** 10.1007/s10955-019-02225-3

**Published:** 2019-02-19

**Authors:** Dorottya Beringer, Ádám Timár

**Affiliations:** Alfréd Rényi Institute of Mathematics, Hungarian Academy of Sciences, Reáltanoda u. 13-15, Budapest, 1053 Hungary

**Keywords:** Random graphs, Controllability, Matching ratio, Benjamini–Schramm convergence, Scale-free networks, Random configuration model

## Abstract

There is an important parameter in control theory which is closely related to the directed matching ratio of the network, as shown in the paper of Liu et al. (Nature 473:167–173, 2011). We give proofs of two main statements of Liu et al. (2011) on the directed matching ratio, which were based on numerical results and heuristics from statistical physics. First, we show that the directed matching ratio of directed random networks given by a fix sequence of degrees is concentrated around its mean. We also examine the convergence of the (directed) matching ratio of a random (directed) graph sequence that converges in the local weak sense, and generalize the result of Elek and Lippner (Proc Am Math Soc 138(8):2939–2947, 2010). We prove that the mean of the directed matching ratio converges to the properly defined matching ratio parameter of the limiting graph. We further show the almost sure convergence of the matching ratios for the most widely used families of scale-free networks, which was the main motivation of Liu et al. (2011).

## Introduction and Results

Liu et al. [10] examined the controllability of both real networks and network models. The models that were most relevant to them are the so-called scale-free networks, which are known to exhibit several characteristics, such as a power-law degree decay, of the networks observed in real-world applications. Informally, the controllability parameter of a network is defined as the minimum number $N_D$ of nodes needed to control a network, e.g., the number of nodes that can shift molecular networks of the cell from a malignant state to a healthy state. They showed that the proportion $n_D=N_D/|V(G)|$ of nodes needed to control a finite network *G* equals one minus the relative size of the maximal directed matching (directed matching ratio, see Definition 1). This allows one to prove results on $n_D$ by proving the corresponding statement for the directed matching ratio. In the paper [10] it was also observed that the matching ratio is mainly determined by the degree sequence of the graph; more precisely, if the edges are randomized in a way that does not change the degrees, then the matching ratio does not change significantly. Furthermore, for the most widely used families of scale-free networks, the directed matching ratio converges to a constant. These two latter statements were based on numerical results, and for the last one methods from statistical physics were also used. In this paper we give rigorous mathematical proofs of these results on the directed matching ratio.

Our first theorem gives a quantitative result on the observation that the matching ratio is concentrated if we randomize the edges of a directed graph in a way that does not change the in- and out-degrees. Furthermore, we show that a similar concentration holds if we randomize the edges in such a way that preserves the total degrees but can alter the number of edges pointing to or from the particular vertices. For the definition of the random configuration model used in the next theorem, see Sect. 1.4.

### Theorem 1

(Concentration of the matching ratio) Consider sequences of in- and out-degrees $d_1^+,\dots ,d_n^+$ and $d_1^-,\dots ,d_n^-$ with $\sum _{j=1}^nd_j^-=\sum _{j=1}^nd_j^+$ and let $d_j=d_j^++d_j^-$. Denote by $e(G):=\sum _{j=1}^nd_j^-$ the number of the edges.

(1) Let *G* be a random directed graph on *n* vertices given by the random configuration model conditioned on the event that the in- and out-degrees are $d_1^+,\dots ,d_n^+$ and $d_1^-,\dots ,d_n^-$, respectively. Then the directed matching ratio *m*(*G*) of *G* satisfies

$$\begin{aligned} \mathrm {\mathbb {P}}\left( |m(G)-{\mathbb {E}}(m(G))|>\varepsilon \right) \le 2\exp \left\{ -\frac{\varepsilon ^2n^2}{8e(G)} \right\} . \end{aligned}$$

(2) Let *G* be a random directed graph on *n* vertices given by the random configuration model conditioned on the event that the total degrees of the vertices are $d_1,\dots , d_n$. Then the directed matching ratio *m*(*G*) of *G* satisfies

$$\begin{aligned} \mathrm {\mathbb {P}}\left( |m(G)-{\mathbb {E}}(m(G))|>\varepsilon \right) \le 2\exp \left\{ -\frac{\varepsilon ^2n^2}{8e(G)} \right\} . \end{aligned}$$

Consider random graph models which ensure that the number of edges is $o(n^2)$ with probability tending to 1. Theorem 1 shows that for graph sequences given by such models, we have a strong concentration of the matching ratio around its mean in the re-randomized graphs with high probability. In particular, the above condition is satisfied if there is a uniform finite bound on the mean of the degrees with probability tending to 1. Erdős–Rényi graphs with parameters (*n*, *c* / *n*) or graphs given by the random configuration model with degree distribution $\xi$ with ${\mathbb {E}}\xi <\infty$ have this property.

Our second result proves the convergence of the matching ratio in the most common families of directed networks. See Definitions 3 and 4 and Remark 2 for the notion of graph convergence and Definition 5 for unimodularity. For the graph models used in the theorem see Sect. 1.4.

### Theorem 2

(Almost sure convergence of the matching ratio for scale-free graphs) (1) Let $G_n$ be a sequence of random (directed) finite graphs that converges to a random rooted (directed) graph (*G*, *o*) in the local weak sense. Then ${\mathbb {E}}(m(G_n))$ converges, namely

$$\begin{aligned} \lim _{n\rightarrow \infty }{\mathbb {E}}(m(G_n))=\sup _M\mathrm {\mathbb {P}}_G\big (o\in V^{(+)}(M)\big ), \end{aligned}$$

where the supremum is taken over all (directed) matchings *M* of *G* such that the distribution of (*G*, *M*, *o*) is unimodular, and $V^{(+)}(M)$ denotes the set of endpoints of the edges in *M* in the undirected case and the set of the heads of the edges of *M* in the directed case.

(2) Let $G_n$ be a sequence of undirected finite graphs defined on a common probability space that converges almost surely in the local weak sense and let  be a sequence of random directed graphs obtained from $G_n$ by giving each edge a random orientation independently. Then  converges almost surely to the constant .

(3) Let $G_n$ be the sequence of random directed graphs given by the preferential attachment rule. Then $m(G_n)$ converges almost surely to the constant $\lim _{n\rightarrow \infty }{\mathbb {E}}(m(G_n))$.

The paper is organized as follows. In Sect. 1.1 we state our main results in the language of control theory and summarize our contribution to the main topics of the paper [10] of Liu, Slotine and Barabási.

In Sect. 2 we present the proof of Theorem 1.

We prove Theorem 2 in Sect. 3. In Sect. 3.1 we prove part (1): in Theorem 6 we show the convergence of the mean of the matching ratio. It was proven in [8] that the limit of the matching ratio of local weak convergent sequences of *deterministic* finite graphs with a uniform bound on the degrees exists. Bordenave et al. [6] removed the bounded degree assumption and gave a formula on the value of the limit of the matching ratio. We still need the context of random directed graphs, hence could not apply their result directly. We proceeded through an alternative definition of the matching ratio of the limit object, which looks more natural in our setting. However, the formula in [6] for the matching ratio of the limit can be adapted, to obtain quantitative results on the asymptotic value of the directed matching ratio or controllability parameter of large random networks.

In Sect. 3.2.1 we prove the results on the matching ratio that imply part (2). This part could also be proven using the concentration in Theorem 1, but we use another method, based on previous results. We prove that if a sequence of random directed graphs is obtained from a convergent deterministic graph sequence by orienting each edge independently, then it converges almost surely in the local weak sense, see Definition 4. This is our Lemma 5 which is similar to Proposition 2.2 in [7]. As a consequence, we get that for directed graphs obtained from almost sure convergent undirected graph sequences the matching ratios converge almost surely. This result applies for sequences given by the random configuration model or Erdős–Rényi random graphs.

In Sect. 3.2.2 we prove the result that implies part (3) of Theorem 4. The method used in Sect. 3.2.1 does not apply to the preferential attachment graphs (we cannot start from an a priory almost sure convergence of the undirected graph sequence) hence we needed a different method.

We note that one can approach the directed matching ratio through an algorithmic point of view, as initiated in [11] via the application of the Karp–Sipser algorithm. We do not pursue this direction in the present paper, but preliminary investigations have been started with E. Csóka.

### Our Contribution to Controllability of Complex Networks

We devote this subsection to the presentation of our results in a network theoretical language. We also highlight the relevance of the present paper for the network theoretical research community; in particular, as a follow-up of the work [10] of Liu, Slotine and Barabási.

Denote by $n_D$ the proportion of the minimum number of driver nodes needed to control the network *G* to the number of nodes, as defined in [10]. Our Theorems 1 and 2 translate to Theorems 3 and 4 by $n_D(G)=1-m(G)$.

Previous results in [8] and [6] on the matching ratio and our Theorem 4 imply that the parameter $n_D$ is a quantity determined by the local structure of the graph. It is a natural question whether even the most local structure, namely the degrees of the vertices, determines this parameter. Liu, Slotine and Barabási approached this question through simulation: in their paper [10] some particular networks are taken from the real world, and then the edges are randomly reshuffled while keeping all in- and out-degrees unchanged. More precisely, the randomization used in [10] generates a random graph ${\bar{G}}$ with the same distribution as given by the random configuration model with the fixed sequence of in- and out-degrees, conditioned to be a simple directed graph (see Sect. 1.4 for the definition of the model). The parameter $n_D$ of several real-world networks was compared with the average of this parameter over the randomized copies. (Generating a large number of random copies provides an average $n_D$ that is close to the expectation of the random $n_D$ by the Law of Large Numbers.) Their results suggested that for random graphs given by the above model, the degrees essentially determine $n_D$.

The first part of our Theorem 3 shows that for a random directed graph *G* given by the random configuration model with fixed in- and out-degrees, the difference between the parameter $n_D$ of the random *G* and the expectation of $n_D$ is small with large probability. This result confirms the phenomenon observed in [10]: with large probability, the parameter $n_D$ of a randomized graph is close to the average, even with the randomization used in [10], see Corollary 1. Our theorem compared with the results in [10] also shows that the examined real-world networks could have been produced by a process that results in a random graph with distribution given by the random configuration model with fixed in- and out-degrees. Our theorem can also be used to substitute generating random networks in order to show concentration.

The second part of Theorem 3 shows that even the total degrees of the network encode the controllability parameter $n_D$ if we assume that the orientation of the edges are independent. In this model we only fix the total degrees of the vertices, generate the random undirected graph, and orient each edge independently. We proved that for this model the parameter $n_D$ of the random graph *G* is close to the expectation of $n_D$ with large probability. We can use this theorem only for graphs generated by the above algorithm. Such graphs have the property that the in- and out-degrees have the same distribution.

#### Theorem 3

(Concentration of the controllability parameter) Consider a sequence of in- and out-degrees $d_1^+,\dots ,d_n^+$ and $d_1^-,\dots ,d_n^-$ with $\sum _{j=1}^nd_j^-=\sum _{j=1}^nd_j^+$ and let $d_j=d_j^++d_j^-$. Denote by $e(G):=\sum _{j=1}^nd_j^-$ the number of the edges.

(1) Let *G* be a random directed network on *n* vertices given by the random configuration model conditioned on the event that the in- and out-degrees are $d_1^+,\dots ,d_n^+$ and $d_1^-,\dots ,d_n^-$, respectively. Then the controllability parameter $n_D(G)$ of *G* satisfies

$$\begin{aligned} \mathrm {\mathbb {P}}\left( |n_D(G)-{\mathbb {E}}(n_D(G))|>\varepsilon \right) \le 2\exp \left\{ -\frac{\varepsilon ^2n^2}{8e(G)} \right\} . \end{aligned}$$

(2) Let *G* be a random directed network on *n* vertices given by the random configuration model conditioned on the event that the total degrees of the vertices are $d_1,\dots , d_n$. Then the controllability parameter $n_D(G)$ of *G* satisfies

$$\begin{aligned} \mathrm {\mathbb {P}}\left( |n_D(G)-{\mathbb {E}}(n_D(G))|>\varepsilon \right) \le 2\exp \left\{ -\frac{\varepsilon ^2n^2}{8e(G)} \right\} . \end{aligned}$$

#### Corollary 1

(Concentration of the controllability parameter in conditioned graphs) Let the random directed graph *G* defined as in Part (1) of Theorem 3 and let ${\bar{G}}$ have the same distribution, but conditioned to be a simple graph. Then the controllability parameter of ${\bar{G}}$ is concentrated around ${\mathbb {E}}(n_D(G))$:

$$\begin{aligned}&\mathrm {\mathbb {P}}\Big (\Big |n_D\big ({\bar{G}}\big )-{\mathbb {E}}(n_D(G))\Big |>\varepsilon \Big )\\&\quad =\frac{\mathrm {\mathbb {P}}\left( |n_D(G)-{\mathbb {E}}(n_D(G))|>\varepsilon , G \text { is simple}\right) }{\mathrm {\mathbb {P}}(G \text { is simple})}\\&\quad \le \frac{2}{\mathrm {\mathbb {P}}(G \text { is simple})}\exp \left\{ -\frac{\varepsilon ^2n^2}{8e(G)} \right\} . \end{aligned}$$

Our Theorem 4 confirms the observations of [10] that the parameter $n_D$ converges for some particular network models when the size of the networks tends to infinity. In fact, Part (1) shows that the expectation of the parameter $n_D$ converges for any sequence of directed graphs that converges in the local weak sense. There are several network models that are known to perform this type of convergence, e.g., Erdős–Rényi random graphs, random *d* regular graphs, networks given by the random configuration model, preferential attachment graphs (see Sect. 1.4 for the definitions and convergence). Part (2) and (3) implies that the graph sequences listed above have an even stronger property: their controllability parameter $n_D$ converges almost surely to a constant. We present these results in Corollaries 4, 3, 2 in Sect. 3.2.1 and Part (3) of Theorem 4. For the first three networks one can derive the limit of $n_D(G_n)=1-m(G_n)$ using the formula of Theorem 2 in [6], see Corollaries 4, 2 and Remark 6 in Sect. 3.2.1.

It was observed in [10] that the nodes with low degree are more likely to be driver nodes, i.e., nodes with in-degree zero in the maximal size directed matching. The method of the proof of the first part of our Theorem 4 shows that this feature of the driver nodes follows naturally from the construction of a maximal size matching: the nodes with higher degrees are more likely to be matched.

#### Theorem 4

(Almost sure convergence of the controllability parameter for scale-free graphs) (1) Let $G_n$ be a sequence of random directed finite graphs that converges to a random rooted graph (*G*, *o*) in the local weak sense. Then ${\mathbb {E}}(n_D(G_n))$ converges, namely

$$\begin{aligned} \lim _{n\rightarrow \infty }{\mathbb {E}}(n_D(G_n))=\inf _M\mathrm {\mathbb {P}}_G\big (o\notin V^{+}(M)\big ), \end{aligned}$$

where the infimum is taken over all directed matchings *M* of *G* such that the distribution of (*G*, *M*, *o*) is unimodular, and $V^+(M)$ denotes the set of the heads of the edges in *M*.

(2) Let $G_n$ be a sequence of undirected finite graphs defined on a common probability space that converges almost surely in the local weak sense and let  be a sequence of random directed graphs obtained from $G_n$ by giving each edge a random orientation independently. Then  converges almost surely to the constant .

(3) Let $G_n$ be the sequence of random directed graphs given by the preferential attachment rule. Then $n_D(G_n)$ converges almost surely to the constant $\lim _{n\rightarrow \infty }{\mathbb {E}}(n_D(G_n))$.

### Notations

We always consider locally finite graphs, with directed or undirected edges. We allow multiple edges and loops. Given the graphs *G* and $G'$ we denote by $G\simeq G'$ and $(G,o)\simeq (G',o)$ that the two graphs are isomorphic and rooted isomorphic, respectively. We write $\deg _G x$ for the degree of a vertex *x* in a graph *G*. If the graph *G* is directed then denote by $\deg ^{in}_G x$ and $\deg ^{out}_G x$ the in- and out-degree of the vertex *x*. Given a directed edge $e=(x,y)$ we call *x* the *tail* and *y* the *head* of the edge. Given a set *F* of edges let *V*(*F*) be the set of vertices that are incident to an edge in *F*. Let $V^-(F)$ and $V^+(F)$ be the set of the tails and the heads of the edges in *F*, respectively. Let $B_G(x,n):=\{y\in V(G): {{\text {dist}}}_G(x,y)\le n\}$ be the ball of radius *n* around a vertex *x* in the graph *G* induced by the graph metric. Given a (multi)set *F* (of edges or vertices) we denote by |*F*| the number of elements of the set (counted with multiplicity). Let [*n*] be the set $\{1,\dots ,n\}$. Given a random graph *G* we denote by $\mathrm {\mathbb {P}}_G$ the probability with respect to its distribution.

### Directed Matchings and Graph Convergence

First we define directed matchings and the matching ratio of directed graphs which are closely related to the controllability of the network.

#### Definition 1

(*Directed matching and directed matching ratio*) A *directed matchingM* of a directed graph *G* is a subset of the edges such that the in- and out-degrees in the subgraph induced by *M* are at most one. The *directed matching ratio* of the finite directed graph *G* is $m(G):=\frac{|V^+(M_{max}(G))|}{|V(G)|}=\frac{|M_{max}(G)|}{|V(G)|}$, where $M_{max}$ is a maximal size directed matching of *G*. For undirected finite graphs *G* we define the matching ratio as $m(G):=\frac{|V(M_{max}(G))|}{|V(G)|}=\frac{2|M_{max}(G)|}{|V(G)|}$, where $M_{max}$ is a maximal size matching of *G*.

For possibly disconnected graphs (for instance Erdős–Rényi graphs or graphs defined by the random configuration model, see Sect. 1.4), there is another natural way to define the directed matching ratio. Viewing them as a unimodular random graph, one takes a uniformly chosen random root, and only keeps the *connected component* of this root. Then one could define the matching ratio as the size of the maximal matching of this component divided by the size of the component. Contrary to connected graphs, this later definition can give a random variable even if we consider deterministic but disconnected graphs. The reason of using Definition 1 in this paper is coming from our motivating applications in controllability. In a finite directed graph the minimum number of nodes needed to control the network equals the number of vertices that have in-degree 0 in a maximal directed matching $M_{max}$ (which equals $|V(G)|-|M_{max}(G)|$); see [10]. We are thus interested in the directed matching ratio *m*(*G*) of a finite directed graph *G* provided by Definition 1, which takes the proportion of vertices of the (possibly disconnected) network that are not needed to control the dynamics of the system.

In this section we describe the relationship between the matching ratio of directed and undirected graphs. We further define the local weak convergence of graph sequences.

#### Definition 2

(*Bipartite representation of a directed graph*) The *bipartite representation* of a directed graph $G=(V,E)$ is the bipartite graph $G'=(V^-,V^+,E')$ with $V^-=\{v^-: v\in V\}$, $V^+=\{v^+:v\in V\}$ and $E':=\{\{v^-,w^+\}:(v,w)\in E\}$.

#### Remark 1

There is a natural bijection between the directed matchings of *G* and the matchings of $G'$ which preserves the size of the matching, namely if *M* is a directed matching of *G* then $M\mapsto M'=\{\{v^-,w^+\}: (v,w)\in M\}$. Furthermore, *M* is a directed matching of maximal size if and only if $M'$ is a maximal size matching of $G'$. It follows that $m(G)=m(G')$.

Recall, that a matching *M* of *G* has maximal size if and only if there is no augmenting path in *G* for *M*. By an augmenting path of length *k* we mean a sequence of disjoint vertices $(v_0,\dots ,v_{2k+1})$ such that $\{v_{2j-1},v_{2j}\}\in M$ for $j\in [k]$, $\{v_{2j},v_{2j+1}\}\notin M$ for $j\in \{0,\dots ,k\}$ and $\deg _Mv_0=\deg _Mv_{2k+1}=0$.

We examine sequences of networks that have bounded average degrees. Benjamini and Schramm [2] introduced a notion of convergence for such graph sequences:

#### Definition 3

(*Local weak convergence of graphs*) We say that the sequence $(G_n,o)$ of locally finite random rooted graphs converge to the locally finite connected random graph (*G*, *o*) in the *local weak sense* if for any positive integer *r* and any finite rooted graph (*H*, *o*) we have $\mathrm {\mathbb {P}}\big (B_{G_n}(o,r)\simeq (H,o)\big )\rightarrow \mathrm {\mathbb {P}}\big (B_{G}(o,r)\simeq (H,o)\big )$.

#### Remark 2

By the local weak convergence of a sequence $G_n$ of non-rooted finite graphs we always mean the convergence of the sequence with a root chosen uniformly at random among the vertices.

For some of the examined graph sequences the following stronger property holds as well:

#### Definition 4

(*Almost sure local weak convergence*) Let $G_n$ be a sequence of finite (directed) random graphs defined on a common probability space (if we do not specify the probability space, then we always consider the product space). We say that $G_n$*converges almost surely in the local weak sense* if almost every realizations of the sequence $(G_n)$ (with respect to the probability measure of the common probability space) satisfy that the sequence of the deterministic graphs converges in the local weak sense.

Finite random graphs with a uniformly chosen root and random rooted graphs that are local weak limits of (random) finite graphs, satisfy the so-called Mass Transport Principle, see [2], Sect. 3.2. The class of graphs that obeys this principle are called *unimodular* graphs.

#### Definition 5

(*Unimodular graphs*) A random rooted (directed, labeled) graph (*G*, *o*) is called unimodular if it obeys the Mass Transport Principle: for every measurable real valued function *f* on the class of locally finite graphs with an ordered pair of vertices that satisfies $f(G,x,y)=f(\gamma G, \gamma x, \gamma y)$ for every $\gamma \in Aut(G)$ the following holds:

$$\begin{aligned} {\mathbb {E}}\left( \sum _{x\sim o}f(G,o,x)\right) ={\mathbb {E}}\left( \sum _{x\sim o}f(G,x,o)\right) . \end{aligned}$$

Directed matchings and hence the matching ratio of a finite directed graph *G* can be examined using the bipartite representation $G'$ as mentioned in Remark 1. In the next proposition, we analyze the relationship between a convergent graph sequence and its bipartite representation.

#### Proposition 1

If a sequence $G_n$ of random directed graphs converges to the random rooted directed graph (*G*, *o*), then the bipartite representations $G'_n$ converge to $(G',o')$, where $G'$ is the bipartite representation of *G* with root $o'$ being $o^-$ or $o^+$ with probability 1/2–1/2.

The converse does not hold: the convergence of the sequence of bipartite representations $G'_n$ does not imply the convergence of $G_n$. In fact, there are different random directed rooted graphs $(G_1,o_1)$ and $(G_2,o_2)$ that are limits of sequences of finite random rooted graphs such that $(G'_1,o'_1)$ is isomorphic to $(G'_2,o'_2)$.

#### Proof

Denote the distribution of $B_{G_n}(o,r)$ and $B_{G}(o,r)$ in the space of locally finite rooted directed graphs by $\mu _{n,r}$ and $\mu _r$, respectively. Similarly, denote the distribution of $B_{G'_n}(o',r)$ and $B_{G'}(o',r)$ in the space of locally finite rooted graphs by $\mu '_{n,r}$ and $\mu '_r$, respectively. The random uniform root $o'$ of a bipartite representation $G'_n$ of a finite directed graph $G_n$ is $o^-$ or $o^+$ with probability 1/2–1/2, where *o* is a uniform random root of *G*. It follows that $\mu '_{n,r}=1/2\mu '_{n,r,o^-}+1/2\mu '_{n,r,o^+}$, where $\mu '_{n,r,o^-}$ and $\mu '_{n,r,o^+}$ are the distributions of $B_{G'_n}(o^-,r)$ and $B_{G'_n}(o^+,r)$, respectively. The first statement of the remark follows.

An example to the second statement is the following. Let $G_1$ be the graph with vertex set $V(G_1)={\mathbb {Z}}$ and edge set $E(G_1)=\{(2k,2k-1), (2k,2k+1):k\in {\mathbb {Z}}\}$, i.e., the usual graph of ${\mathbb {Z}}$ with an alternating orientation to the edges. Let the random root $o_1$ be 2*k* or $2l-1$ for some $k,l\in {\mathbb {Z}}$ with probability 1/2–1/2 (the isomorphism class of $(G_1,o)$ does not depend on the actual choice of the integers *k* and *l*). This graph is the limit of the cycles $C_{2n}$ with 2*n* vertices and edges with alternating orientations. Let $G_2$ be the one-point graph without edges with probability 1/2 and with probability 1/2 let $G_2$ be the infinite regular tree with in- and out-degrees 2. This graph is the limit of the sequence of random graphs on *n* vertices where with probability 1/2 there are no edges and with probability 1/2 the graph is uniformly randomly chosen from the set of graphs on *n* vertices with all in- and out-degrees 2. Then $(G'_1,o'_1)$ and $(G'_2,o'_2)$ are both isomorphic to the random graph that is the one-point graph without edges or ${\mathbb {Z}}$ with probability 1/2–1/2. $\square$

### Canonical Network Models and Their Limits

Some of the examined graph sequences converge to the so-called unimodular Galton–Watson tree.

#### Definition 6

(*Unimodular Galton–Watson tree*) Let $\xi$ be a non-negative integer valued random variable with ${\mathbb {E}}\xi <\infty$. The unimodular Galton–Watson tree with offspring distribution $\xi$ (denoted by $UGW(\xi )$) is a random rooted tree with root *o*. We say that a vertex *y* is the child of the vertex *x* if they are adjacent and ${{\text {dist}}}(y,o)={{\text {dist}}}(x,o)+1$. The graph $UGW(\xi )$ is given by the following recursive definition:

- The probability that *o* has $k\ge 0$ children is $\mathrm {\mathbb {P}}(\xi =k)$.
- For each vertex *x* the probability that *x* has $k\ge 0$ children is $\frac{(k+1)\mathrm {\mathbb {P}}(\xi =k+1)}{{\mathbb {E}}\xi }$.

Let the directed unimodular Galton–Watson tree  be the random rooted directed graph obtained from $UGW(\xi )$ by orienting each edge independently.

Now we present the network models examined in this paper. For each model first we define the non-directed model and present the known results on the local weak limit of the sequence, then we give the definition of the directed versions and the local weak limit of them.

**Erdős–Rényi Random Graphs**

The Erdős–Rényi random graphs ${\mathcal {G}}_{n,c/n}$ are defined in the following way: consider the complete graph on *n* vertices and keep each edge with probability *c* / *n* or delete it with probability $1-c/n$ independently from each other. The resulting random graph is ${\mathcal {G}}_{n,c/n}$.

The local weak limit of ${\mathcal {G}}_{n,c/n}$ is *UGW*(Poi(*c*)), that is the Galton–Watson tree with Poisson(*c*) offspring distribution. In fact, for almost every realization of the sequence ${\mathcal {G}}_{n,c/n}$, that sequence of deterministic graphs converges to *UGW*(Poi(*c*)) as well, see Theorem 3.23 in [5].

We define the directed Erdős–Rényi random graphs  by orienting each edge of ${\mathcal {G}}_{n,c/n}$ uniformly at random independently for the edges. The local weak limit of this sequence is .

**Random** *d* **-Regular Graphs**

Let $G_n$ be the random graph chosen uniformly at random from the set of graphs on the vertex set [*n*] with all degrees equal *d*. It is standard, that the local weak limit of $G_n$ as $n\rightarrow \infty$ is the infinite *d*-regular tree ${\mathbb {T}}_d$. In fact, the random graphs $G_n$ converge almost surely to ${\mathbb {T}}_d$. This follows from the almost sure convergence of the more general class of graphs given by the random configuration model.

There are two natural ways to define random directed regular graphs. The first one is if $G_n$ is a uniformly chosen directed graph on [*n*] such that each vertex has in- and out-degrees *d*. The local weak limit is a regular tree with in- and out-degrees *d*. The second way to define directed graphs $G_n$ is if we choose a uniform random non-directed *d*-regular graph on [*n*] and orient each edge uniformly at random independently from each other. This model is a special case of the random configuration model defined in the sequel. The limit of that graph sequence is the *d*-regular tree with independently oriented edges.

The next two graphs have become increasingly important in applications, because they grab important characteristics of real-world networks (scale-free networks). This is the reason why in [10], which was motivated by applications of controllability, these graphs were studied.

**Random Configuration Model**

We fix a non-negative integer valued probability distribution $\xi$. We define the (multi)graph $G_n$ in the following way: let $\xi _1,\dots ,\xi _n$ be i.i.d. variables with distribution $\xi$. Given $\xi _1,\dots ,\xi _n$ let ${\mathcal {E}}:=\{(k,j):k\in [n], j\in [\xi _k]\}$ be the set of the *half-edges*. Let *R* be a uniform random perfect matching of the set ${\mathcal {E}}$ (if $|{\mathcal {E}}|$ is odd, then put off one half-edge uniformly at random before choosing a perfect matching). Then *R* defines the random (multi)graph $G_n=G_n(R)$ on [*n*]. One can also consider the random graph ${\bar{G}}_n$ by conditioning the random configuration model to give a simple graph.

If ${\mathbb {E}}(\xi ^2)<\infty$, then $G_n$ converge to $UGW(\xi )$ in the local weak sense (see Theorem 3.15 in [5]). Furthermore, if ${\mathbb {E}}(\xi ^p)<\infty$ with some $p>2$, then for almost every realization of the sequence $G_n$, the local weak limit of that deterministic graph sequence is $UGW(\xi )$, and the same holds for ${\bar{G}}_n$; see Theorem 3.28 in [5] or Theorem 7.

If we want to define a directed graph, then we orient each edge uniformly at random independently from the other edges. We get the same distribution if after fixing the degree sequence $\xi _1,\dots ,\xi _n$ we select a subset ${\mathcal {E}}_T\subseteq {\mathcal {E}}$ of size $\lfloor |{\mathcal {E}}|/2\rfloor$ uniformly at random. Then we set $\xi _k^-:=|\{j\in [\xi _k]: (k,j)\in {\mathcal {E}}_T\}|$, $\xi _k^+:=\xi _k-\xi _k^-$ and we denote by ${\mathcal {T}}:=\{(k,j,-):k\in [n], j\in [\xi _k^-]\}$ the set of the tail-type half-edges and by ${\mathcal {H}}:=\{(k,j,+):k\in [n], j\in [\xi _k^+]\}$ the set of the head-type half-edges. Let ${\mathcal {N}}$ be the set of the perfect matchings of ${\mathcal {T}}$ to ${\mathcal {H}}$ and denote by *N* a uniform random element of ${\mathcal {N}}$. Then *N* defines the random directed graph $G_n=G_n(N)$ on the vertex set [*n*].

**Preferential Attachment Graphs**

The notion of preferential attachment graphs was introduced by Barabási and Albert in [1] and the precise construction was given by Bollobás and Riordan in [4]. There are several versions of the definition of this family of random graphs which have turned out to be asymptotically the same: they all converge to the same infinite limit graph; see [3]. Although in the original definitions the preferential attachment graphs are not directed, there is a natural way to give each edge an orientation and these orientations extend to the limit graph as well.

We will use the following definition from [3] completed with the natural orientation of the edges: fix a positive integer *r* and $\alpha \in [0,1)$. For each *n* the random graph $G_n=G_{r,\alpha ,n}^{PA}$ is a graph on the vertex set [*n*] defined by the following recursion: let $G_{0}$ be the graph with one vertex and no edges. Given $G_{n-1}$ we construct $G_{n}$ by adding the new vertex *n* and *r* new edges with tails *n*. We choose the heads $w_1,\dots w_r$ of the new edges independently from each other in the following way: with probability $\alpha$ we choose $w_j$ uniformly at random among $[n-1]$, and with probability $1-\alpha$ we choose $w_j$ proportional to $\deg _{G_{n-1}}$. Note that each vertex except the starting vertex has out-degree *r* and each vertex has a random in-degree with mean converging to *r*.

Berger et al. proved in [3] that the local weak limit of $G_{r,\alpha ,n}^{PA}$ as $n\rightarrow \infty$ is the Pólya-point graph with parameters *r* and $\alpha$. This graph is a unimodular random infinite tree with directed edges; see [3], Sect. 2.3 for the definition.

## Concentration of the Matching Ratio in Randomized Networks—Proof of Theorem 1

In this section we prove Theorem 1, which gives a quantitative version of the following experimental observation of Liu et al. in [10]: if we consider a large directed graph, and randomize the edges in such a way that does not change the in- and out-degrees of the graph, then the matching ratio does not alter significantly. Part (1) of Theorem 1 shows the concentration for randomized graphs with the in- and out-degrees left unchanged. This is the result that was observed through simulations in [10]. Part (2) of the theorem shows that a very similar concentration phenomenon holds even after a randomization that can change the in- and out-degrees but keeps the total degrees unchanged for every vertex. In particular, Theorem 1 shows that if a graph sequence satisfies that the mean of the degree sequence is bounded with probability tending to 1 (as $n\rightarrow \infty$), then the directed matching ratios of the graphs with randomized edges are concentrated around their mean.

First we need a lemma that shows that modifying a (directed) graph just around a few vertices cannot alter the size of the maximal matching too much.

### Lemma 1

Adding some new edges with a common endpoint to an undirected finite graph or adding edges with a common head (or common tail) to a directed finite graph can increase the size of the maximal matching by at most one.

### Proof

For directed graphs the statement follows from the undirected case, using the bipartite representation (see Definition 2). For undirected graphs let *F* be the set of new edges with common endpoint *x* and let $G_2$ be the graph with vertex set *V*(*G*) and edge set $E(G_2)=E(G)\cup F$. If $M_2$ is a maximal size directed matching of $G_2$, then there is at most one edge in $M_2\cap F$ by the definition of the matching. Then $M_2\setminus F$ is a matching of *G*, hence $|M_{max}(G)|\ge |M_2|-1$. $\square$

Before proving the proposition, we state a version of the Azuma–Hoeffding inequality (see [13], Theorem 13.2), that we will use in this paper.

### Theorem 5

(Azuma–Hoeffding inequality) Let $X_1,\dots ,X_n$ be a series of martingale differences. Then

$$\begin{aligned} \mathrm {\mathbb {P}}\left( \sum _{k=1}^n X_k>\varepsilon \right) \le \exp \left\{ -\frac{\varepsilon ^2}{2\sum _{k=1}^n \Vert X_k\Vert ^2_{\infty }}\right\} . \end{aligned}$$

The proof of Theorem 1 uses similar methods to that of Corollary 3.27 in [5], which implies the concentration of matching ratio for undirected graphs.

### Proof of Theorem 1

We prove both parts of the theorem in the following way: we define random variables $X_k$, $k\in [e(G)]$ which form a series of martingale differences and satisfy $\sum _{k=1}^{e(G)}X_k=n\big (m(G)-{\mathbb {E}}(m(G))\big )$. We will show that there is an almost sure bound $|X_k|\le 2$, hence we have by the Azuma–Hoeffding inequality

$$\begin{aligned} \mathrm {\mathbb {P}}\left( |m(G(N))-{\mathbb {E}}(m(G(N)))|>\varepsilon \right)&=\mathrm {\mathbb {P}}\left( |X_1+\dots +X_{e(G)}|>\varepsilon n \right) \\&\le 2\exp \left\{ -\frac{(\varepsilon n)^2}{2\sum _{k=1}^{e(G)} \Vert X_k\Vert _\infty ^2} \right\} \\&\le 2\exp \left\{ -\frac{\varepsilon ^2n^2}{8e(G)} \right\} . \end{aligned}$$

*Part (1)* Recall the second definition of the directed random configuration model from Sect. 1.4, conditioned on the fixed sequences of in- and out-degrees. Denote by *N* a uniform random element of the set ${\mathcal {N}}$ of perfect matchings of ${\mathcal {T}}$ to ${\mathcal {H}}$. For a half-edge $h=(i,j,\pm )\in {\mathcal {T}}\cup {\mathcal {H}}$ let *N*(*h*) be the pair of the half-edge *h* by the matching *N*. Let $\{h_i:i\in [e(G)]\}$ be an enumeration of ${\mathcal {T}}$ and denote by $N[k]:=\{(h_i,N(h_i))\in N: i\in [k]\}$ the partial matching that consists of the pairs of half-edges of *N* with the first *k* tails. Let ${\mathcal {F}}_0$ be the trivial $\sigma$-algebra, let ${\mathcal {F}}_k$ be the $\sigma$-algebra generated by *N*[*k*] and define

1 $$\begin{aligned} X_k:={\mathbb {E}}\left( |M_{max}(G(N))| \, \Big |\, {\mathcal {F}}_k\right) -{\mathbb {E}}\left( |M_{max}(G(N))|\, \Big |\, {\mathcal {F}}_{k-1}\right) . \end{aligned}$$

The variables $X_k$ clearly form a series of martingale differences, and we claim that $|X_k|\le 2$ almost surely for all $k\in [e(G)]$.

Fix an arbitrary partial matching $F_0=\{(h_i, F_0(h_i)):i\in [k]\}$ and let ${\mathcal {N}}_k:=\{S\in {\mathcal {N}}:S[k]=F_0\}$ and ${\mathcal {N}}_{k-1}:=\{S\in {\mathcal {N}}:S[k-1]=F_0[k-1]\}$ be the set of perfect matchings of ${\mathcal {H}}$ to ${\mathcal {T}}$ with $S[k]=F_0$ and $S[k-1]=F_0[k-1]$, respectively. Denote $h':=F_0(h_k)$ and for a configuration $S\in {\mathcal {N}}[k-1]$ let

2 $$\begin{aligned} f(S):=\left( S\setminus \Big \{\big (h_k,S(h_k)\big ),\big (S(h'),h'\big )\Big \}\right) \cup \Big \{(h_k,h'),\big (S(h'),S(h_k)\big )\Big \}. \end{aligned}$$

For each $S\in {\mathcal {N}}_{k-1}$ there is a unique $f(S)\in {\mathcal {N}}_k$ and for each $S'\in {\mathcal {N}}_k$ the size of the set $\{S\in {\mathcal {N}}_{k-1}: f(S)=S'\}$ is equal, namely $e(G)-k=\frac{|{\mathcal {N}}_{k-1}|}{|{\mathcal {N}}_k|}$. We have

3 $$\begin{aligned}&\bigg |{\mathbb {E}}\Big (|M_{max}(G(N))|\, \Big |\, N[k]=F_0\Big )- {\mathbb {E}}\Big (|M_{max}(G(N))|\,\Big |\,N[k-1]=F_0[k-1]\Big ) \bigg | \nonumber \\&\quad = \left| \sum _{S'\in {\mathcal {N}}_k}\frac{|M_{max}(G(S'))|}{|{\mathcal {N}}_k|}-\sum _{S\in {\mathcal {N}}_{k-1}}\frac{|M_{max}(G(S))|}{|{\mathcal {N}}_{k-1}|}\right| \nonumber \\&\quad = \left| \sum _{S\in {\mathcal {N}}_{k-1}}\frac{|M_{max}(G(f(S)))|}{(e(G)-k)|{\mathcal {N}}_{k}|}-\frac{|M_{max}(G(S))|}{|{\mathcal {N}}_{k-1}|}\right| \nonumber \\&\quad \le \sum _{S\in {\mathcal {N}}_{k-1}}\Big | |M_{max}(G(S))|-|M_{max}(G(f(S)))| \Big |\frac{1}{|{\mathcal {N}}_{k-1}|} \end{aligned}$$

For any $S\in {\mathcal {N}}_{k-1}$ the graphs *G*(*S*) and *G*(*f*(*S*)) differ by at most four edges in such a way that the size of the set of the heads of these vertices is at most two. By Lemma 1 we have in this case $\Big |\,|M_{max}(G(S))|-|M_{max}(G(f(S)))|\, \Big | \le 2$ which combined with (3) proves the bound $|X_k|\le 2$.

*Part (2).* Recall the notations and the first definition of the directed random configuration model from Sect. 1.4, conditioned on the fixed sequence of total degrees. Let ${\mathcal {R}}$ be the set of perfect matchings of the set ${\mathcal {E}}$ of half-edges together with an ordering on each matched pair (this ordering gives the orientation of the corresponding edge). Denote by *R* a uniform random element of ${\mathcal {R}}$ and let $\{h,R(h)\}_R=(h,R(h))$ or (*R*(*h*), *h*), i.e., the unique ordered pair containing the half-edge *h* given by the matching *R*. Let ${\mathcal {E}}=\{h_i: i\in [2e(G)]\}$ be an enumeration of the set of half-edges. Let *R*[*k*] be the partial matching that contains the first *k* edges with respect to the ordering given by the minimum of the indices of the two half-edges, i.e., let $R[0]:=\emptyset$ and for $k\ge 1$ let

$$\begin{aligned} R[k]:=R[k-1]\cup \Big \{\{h_i,R(h_i)\}_R:i=\min \{j:h_j\notin {\mathcal {E}}(R[k-1])\}\Big \}, \end{aligned}$$

where ${\mathcal {E}}(R[k-1])$ is the set of half-edges that are paired by the partial matching $R[k-1]$.

Let ${\mathcal {F}}_0$ be the trivial $\sigma$-algebra, let ${\mathcal {F}}_k$ be the $\sigma$-algebra generated by *R*[*k*] and define

$$\begin{aligned} X_k:={\mathbb {E}}\left( |M_{max}(G(R))|\,\Big |\,{\mathcal {F}}_k\right) - {\mathbb {E}}\left( |M_{max}(G(R))|\,\Big |\,{\mathcal {F}}_{k-1}\right) . \end{aligned}$$

We claim that $|X_k|\le 2$ almost surely for all *k*, which implies the statement of the theorem.

Let $F_0$ be any fixed partial matching of ${\mathcal {E}}$ consisting of *k* ordered pairs. We will show that

4 $$\begin{aligned} \Bigg | {\mathbb {E}}\left( |M_{max}(G(R))|\,\Big |\,R[k]=F_0\right) - {\mathbb {E}}\left( |M_{max}(G(R))|\,\Big |\,R[k]=F_0[k-1]\right) \Bigg |\le 2, \end{aligned}$$

which implies the bound $|X_k|\le 2$.

Let ${\mathcal {R}}_{k-1}:=\{S\in {\mathcal {R}}:S[k-1]=F_0[k-1]\}$ and ${\mathcal {R}}_{k}:=\{S\in {\mathcal {R}}:S[k]=F_0\}$. Denote by $(h,h')$ the unique ordered pair in $F_0\setminus F_0[k-1]$ and define

$$\begin{aligned} {\mathcal {S}}:=&\Big \{ (S,S')\in {\mathcal {R}}_{k-1}\times {\mathcal {R}}_k: \\&S(h)=h' \text { and } S\setminus \big \{\{h,h'\}_S\big \}=S'\setminus \{(h,h')\} \\&\text {or }S(h)\ne h'\text { and } |S\bigtriangleup S'|=4 \Big \}. \end{aligned}$$

Note that the pairs $(S,S')\in {\mathcal {S}}$ that satisfy $S(h)\ne h'$,$|S\bigtriangleup S'|=4$, have the properties $S'(S(h))=S(h')$ and

$$\begin{aligned} S\setminus \Big \{\{h,S(h)\}_S,\{h',S(h')\}_S\Big \}=S'\setminus \Big \{(h,h'),\{S(h),S(h')\}_{S'}\Big \}. \end{aligned}$$

For each $S\in {\mathcal {R}}_{k-1}$ with $S(h)=h'$ there is a unique $S'\in {\mathcal {R}}_k$ with $(S,S')\in {\mathcal {S}}$ and for each $S\in \mathcal {R}_{k-1}$ with $S(h)\ne h'$ the cardinality of the set $\{S'\in {\mathcal {R}}_k:(S,S')\in {\mathcal {S}}\}$ is 2. For each $S'\in {\mathcal {R}}_k$ the sets $\{S\in {\mathcal {R}}_{k-1}: S(h)=h', (S,S')\in {\mathcal {S}}\}$ and $\{S\in {\mathcal {R}}_{k-1}:S(h)\ne h',(S,S')\in {\mathcal {S}}\}$ have 2 and $8(e(G)-k)$ elements, respectively. To see that the last claim is true, note that given an $S'\in {\mathcal {R}}_{k}$ one can obtain an $S\in {\mathcal {R}}_{k-1}$ with $S(h)\ne h'$, $(S,S')\in {\mathcal {S}}$ by choosing a pair $(f,f')\in S'\setminus S'[k]$ and let $\{S(h),S(h')\}=\{f,f'\}$. The term 8 comes from the two possible choises of $S(h)=f$ or $f'$ and the ordering of the pairs. Define a function *c* on ${\mathcal {R}}_{k-1}$ by $c(S):=2$ if $S(h)=h'$ and $c(S):=1$ otherwise. Denote $s:=\sum _{(S,S')\in {\mathcal {S}}}c(S)$, which satisfies $s=2|{\mathcal {R}}_{k-1}|=(4+8(e(G)-k))|{\mathcal {R}}_k|$ by the cardinalities of the sets mentioned above. Using these notations we obtain

5 $$\begin{aligned}&\bigg |{\mathbb {E}}\Big (|M_{max}(G(R))|\, \Big |\, R[k]=F_0\Big )- {\mathbb {E}}\Big (|M_{max}(G(R))|\,\Big |\,R[k-1]=F_0[k-1]\Big ) \bigg | \nonumber \\&\quad = \left| \sum _{S'\in {\mathcal {R}}_k}\frac{|M_{max}(G(S'))|}{|{\mathcal {R}}_k|}-\sum _{S\in {\mathcal {R}}_{k-1}}\frac{|M_{max}(G(S))|}{|{\mathcal {R}}_{k-1}|}\right| \nonumber \\&\quad = \left| \sum _{S'\in {\mathcal {R}}_{k}}\frac{\big (4+8(e(G)-k)\big )|M_{max}(G(S'))|}{s}-\sum _{S\in {\mathcal {R}}_{k-1}}\frac{2|M_{max}(G(S))|}{s}\right| \nonumber \\&\quad \le \sum _{(S,S')\in {\mathcal {S}}}\Big | |M_{max}(G(S))|-|M_{max}(G(S'))| \Big |\frac{c(S)}{s} \end{aligned}$$

For any pair $(S,S')\in {\mathcal {S}}$ the graphs *G*(*S*) and $G(S')$ differ by at most four edges in such a way that both of the graphs can be obtained from the same graph by adding at most two edges to it. By Lemma 1 we have in this case $\Big |\,|M_{max}(G(S))|-|M_{max}(G(S'))|\, \Big | \le 2$ which combined with (5) proves the bound $|X_k|\le 2$.

## Convergence of the Matching Ratio—Proof of Theorem 2

The goal of this section is to prove the convergence of the directed matching ratio for convergent sequences of random directed graphs. This convergence is understood in the stronger sense of almost sure convergence, as we will see, but the proof will often proceed through showing convergence in expectation and then concentration. For a fixed deterministic non-directed graph sequence that is locally convergent when a uniform root is taken, the convergence of the matching ratio is proved by Elek and Lippner in [8] if there is a uniform bound on the degrees and by Bordenave, Lelarge and Salez in [6] in the unbounded case. To prove the results of Liu et al. in [10], we need to generalize these results to directed random graphs.

In Sect. 3.1 we use the method of Elek and Lippner to prove Theorem 6 on the convergence of the *expected value* of the directed matching ratio of sequences of random graphs. In Definition 7 we give an extension of the definition of the expected matching ratio to unimodular random rooted graphs. By Theorem 1 in [6] and our Theorem 6 our definition of the expected matching ratio equals twice the parameter $\gamma$ defined in [6].

In Sect. 3.2 we prove the almost sure convergence of the directed matching ratios for the network models defined in Sect. 1.4.

### Convergence of the Mean of the Matching Ratio—Proof of Part (1) of Theorem 2

Elek and Lippner proved that the non-directed matching ratio converges if $G_n$ is a convergent sequence of finite deterministic graphs with uniformly bounded degree; see [8], Theorem 1. There are three properties of our examined models, that do not let us apply this theorem directly: our graphs do not have bounded degrees, and they are directed and random graphs. Although the degrees are not bounded in the examined models of convergent graph sequences, the expected value of the degree of the uniform random root of the random graphs has a uniform bound in each model. In Theorem 6 we prove the convergence of the mean of the matching ratio for convergent sequences of random directed graphs using the method of Elek and Lippner.

One can extend the (expected) matching ratio to the class of unimodular random (directed) graphs in a natural way. For finite random graphs, the following definition gives the expected value of the matching ratio.

#### Definition 7

(*Matching ratio of an infinite graph and unimodular matchings*) Let (*G*, *o*) be a unimodular random (directed) rooted graph. Then the *(expected) matching ratio* of (*G*, *o*) is

$$\begin{aligned}m_E(G,o)=\sup _{M}\mathrm {\mathbb {P}}_G(o\in V^{(+)}(M)),\end{aligned}$$

where $V^{(+)}(M)$ denotes the set of the endpoints of the edges in *M* in the undirected case and the set of the heads of the edges of *M* in the directed case, and the supremum is taken over all random (directed) matchings of *G* such that the distribution of (*G*, *M*, *o*) is unimodular. Matchings with this property will be called *unimodular matchings*.

#### Remark 3

Let (*G*, *o*) be a random directed rooted unimodular graph and let $(G',o')$ be its bipartite representation (see Definition 2). Then Lemma 3 will imply that $m_E(G,o)=m_E(G',o')$.

#### Theorem 6

Let $G_n$ be a sequence of random finite (directed) graphs that converges to the random (directed) rooted graph (*G*, *o*) that has finite expected degree. Then ${\mathbb {E}}(m(G_n))$ converges, namely

$$\begin{aligned} \lim _{n\rightarrow \infty }{\mathbb {E}}(m(G_n))=m_E(G,o). \end{aligned}$$

To prove Theorem 6, we follow the method of [8]. The main differences to that proof come from the lack of uniform bound on the degrees. We will define the matchings *M*(*T*) in Lemma 2 as factor of IID, which helps us handle the case of unbounded degrees. For graphs with unbounded degrees, Lemma 4.1 of [8] does not apply, hence we will have to proceed through Lemma 4.

#### Definition 8

(*Factor of IID*) Let ${\mathcal {G}}_\star$ be the set of the isomorphism classes of locally finite rooted (directed) graphs (*G*, *o*) with ${\mathbb {R}}$-valued labels $\{c_G(v):v\in V(G)\}\cup \{c_G(e):e\in E(G)\}$ on the vertices and edges, equipped with the topology generated by the sets

$$\begin{aligned} \left\{ \begin{array}{r l} (G,o)\in {\mathcal {G}}_\star : &{}\exists \varphi :B_G(o,r)\rightarrow H \text { rooted (dir.) graph homomorphism s.t.} \\ &{}|c_G(a)-c_H(\varphi (a))|<\varepsilon , \forall a\in V(B_G(o,r))\cup E(B_G(o,r)) \end{array}\right\} , \end{aligned}$$

where $\varepsilon >0$, *r* is any positive integer, *H* is any finite rooted (directed) graph with labels $\{c_H(a):a\in V(H)\cup E(H)\}$ on the vertices and edges. A measurable function $f:{\mathcal {G}}_\star \rightarrow {\mathbb {R}}$ is called a *factor*.

Let *G* be a (random directed) graph, let $c:V(G)\rightarrow [0,1]$ be IID uniform random labels on the vertices and let *G*(*c*) be the random labeled graph given by the labels *c*. The collection of random variables $\{X_a=f((G(c),a)):a\in V(G)\cup E(G)\}$ is called a *factor of IID process* if *f* is a factor.

A random subset $M\subseteq E(G)$ is called a *factor of IID (directed) matching* if there is a factor of IID process $(X_a)$ such that an edge *e* is in *M* if and only if $X_e=1$ and *M* is a matching of *G* with probability 1 with respect to the distribution of *G*(*c*).

The factor *f* in the above definition should be interpreted as a rule that gives the new labels of *G*. It follows from the measurability of *f* that a factor of IID process is locally defined in the sense that we can reconstruct the new value of the label at the root up to an arbitrary small error if we know the graph structure and the labels up to a small enough error in the neighborhood of a large enough radius.

We note, that given a unimodular random rooted graph (*G*, *o*) and a factor of IID process $(X_a)$ on *G*, the distribution of the labeled rooted graph $(G,(X_a),o)$ is unimodular as well. In particular, every factor of IID matching *M* of a unimodular graph satisfies that (*G*, *M*, *o*) is unimodular.

#### Lemma 2

(1) For any locally finite graph *G* and any $T>0$ there is a factor of IID matching *M*(*T*) that has no augmenting paths of length at most *T*.

(2) If (*G*, *o*) is a random unimodular rooted graph, then

$$\begin{aligned} \lim _{T\rightarrow \infty }\mathrm {\mathbb {P}}_G\left( o\in V(M(T))\right) =m_E(G,o). \end{aligned}$$

#### Remark 4

The above lemma holds for directed graphs as well: the statements of the lemma remain true for the pre-images of the matchings *M*(*T*) by the bijection defined in Remark 1.

The proof of part (1) of Lemma 2 is similar to that of Lemma 2.2 of [8], but for the sake of completeness we present it here. The main difference is that for graphs with unbounded degrees we cannot define the matchings *M*(*T*) using Borel colorings, which were used in [8]. To handle the case of unbounded degrees we define *M*(*T*) as factor of IID matchings. Our language is also different, although all the claims stated for Borel matchings in [8] hold for factor of IID matchings as well.

We need the following lemma for the proof of part (2) of Lemma 2.

#### Lemma 3

Let (*G*, *o*) be a unimodular random rooted graph. Then if a unimodular matching *M* of *G* satisfies that there are no augmenting paths of length at most *k*, then

$$\begin{aligned} \mathrm {\mathbb {P}}(o\in V(M))\ge m_E(G,o)-1/k. \end{aligned}$$

#### Proof

We show that for every $\varepsilon$ and *k*, any unimodular matching *M* that has no augmenting path of length at most *k* satisfies

6 $$\begin{aligned} \mathrm {\mathbb {P}}(o\in V(M))\ge m_E(G,o)-\varepsilon -1/k. \end{aligned}$$

This implies the statement of the lemma. Let $M_\varepsilon$ be a fixed unimodular matching that satisfies $m_E(G,o)-\mathrm {\mathbb {P}}(o\in V(M_\varepsilon ))\le \varepsilon$. Consider the symmetric difference $M\bigtriangleup M_\varepsilon$, that is a disjoint union of paths and cycles, which alternately consists of edges of *M* and $M_\varepsilon$ by the definition of matchings. We will bound $\mathrm {\mathbb {P}}(o\in V(M_\varepsilon )\setminus V(M))$ from above by 1 / *k*, which implies (6) by

$$\begin{aligned} \mathrm {\mathbb {P}}(o\in V(M))\ge \mathrm {\mathbb {P}}(o\in V(M_\varepsilon ))-\mathrm {\mathbb {P}}(o\in V(M_\varepsilon )\setminus V(M)). \end{aligned}$$

If a vertex *x* of *G* is in $V(M_\varepsilon )\setminus V(M)$, then there is an alternating path consisting of at least $2k+2$ edges in $M\bigtriangleup M_\varepsilon$ starting from *x* with an edge of $M_\varepsilon$ by the assumption on *M*. Define the following mass transport: let $f(x,y,(G,M\bigtriangleup M_\varepsilon ))$ be 1 if $x\in V(M_\varepsilon )\setminus V(M)$ and *y* is at distance at most $k-1$ from *x* in the graph metric induced by $M_\varepsilon \bigtriangleup M$ (there is exactly *k* such *y*, by our previous observation on the alternating path starting from *x*). Let $f(x,y,(G,M\bigtriangleup M_\varepsilon ))$ be 0 otherwise. Note that each vertex receives mass at most 1. The labeled graph $(G,M\bigtriangleup M_\varepsilon ,o)$ is unimodular, hence we have by the Mass Transport Principle that

$$\begin{aligned} k\mathrm {\mathbb {P}}(o\in V(M_\varepsilon )\setminus V(M))&={\mathbb {E}}\left( \sum _{x\in V(G)}f(o,x,(G,M\bigtriangleup M_\varepsilon ))\right) \\&={\mathbb {E}}\left( \sum _{x\in V(G)}f(x,o,(G,M\bigtriangleup M_\varepsilon ))\right) \le 1. \end{aligned}$$

This gives the desired bound on $\mathrm {\mathbb {P}}(o\in V(M_\varepsilon )\setminus V(M))$. $\square$

#### Proof of Lemma 2

We assign to each vertex *x* of *G* a uniform random [0, 1]-label *c*(*x*). First we note that with probability 1 all the labels are different, so we can assume this property. Furthermore, we can decompose each label *c*(*x*) into countably many labels $(c_{i,j}(x))_{i,j=0}^\infty$ whose joint distribution is IID uniform on [0,1]. First we construct partitions ${\mathcal {V}}_T=\{V_{T,j}:j\ge 1\}, T\ge 1$ of *V* such that for each *T* and *j*$\inf \{{{\text {dist}}}(x,y):x,y\in V_{T,j}\}\ge 6T$ holds. Let

$$\begin{aligned} V_{T,1}:=&\left\{ x\in V: c_{T,1}(x)<c_{T,1}(y) \text { for every } y\in B_G(x,6T)\right\} , \\ V_{T,j}:=&\left\{ x\in V \Bigg \backslash \left( \bigcup _{l=1}^{j-1}V_{T,l}\right) : c_{T,j}(x)<c_{T,j}(y) \text { for every } y\in B_G(x,6T)\right\} , \end{aligned}$$

for $j\ge 2$. Since the labels are uniform in [0, 1], we get a partition with probability one.

We define the matchings $M_n(T)$ in the following way. Let $M_0(T)=M(T-1)$ (and the empty matching if $T=1$) and let *k*(*n*) be a fixed sequence that consists of positive integers and contains each of them infinitely many times. To define $M_n(T)$ we improve the matching $M_{n-1}(T)$ in all the balls *B*(*x*, 3*T*) with $x\in V_{T,k(n)}$: we improve using the augmenting path of length at most *T* lying in *B*(*x*, 3*T*) with the maximal sum of $c_{T,0}$-labels of the vertices and we repeat this as long as there are short augmenting paths. The number of vertices in *B*(*x*, 3*T*) that are incident to edges of the matching increases in each step, hence we can make only a finite number of improvements in each ball. Since for all *n* the balls in $\{B(x,3T): \in V_{T,k(n)}\}$ are disjoint, $M_n(T)$ is a well defined matching for every *n* and *T*.

Let *M*(*T*) be the edge-wise limit of $M_n(T)$ as $n\rightarrow \infty$. We claim that *M*(*T*) is well defined and has no augmenting paths of length at most *T*. Indeed, an edge $e=\{x,y\}$ changes its status of being in the matching or not only if there is an improvement in *B*(*x*, 3*T*). Such an improvement increase the number of vertices incident to edges of the matching in *B*(*x*, 3*T*), which is bounded above by the number of vertices in the ball, thus the number of changes is bounded above as well. The lack of short augmenting paths follows trivially from the construction of *M*(*T*).

We note that every factor of IID matching *M* of a unimodular random rooted graph (*G*, *o*) satisfies that (*G*, *M*, *o*) is unimodular, hence Lemma 3 implies the second statement of the lemma. $\square$

Since we do not assume the existence of a uniform bound on the degrees, we need a lemma that plays the role of Lemma 4.1 of [8].

#### Lemma 4

Let (*G*, *o*) be a labeled (directed) unimodular graph with finite expected degree ${\mathbb {E}}(\deg o)<\infty$. Denote the distribution of (*G*, *o*) by $\mu$. Then for any $\varepsilon >0$ and any *n* there is a $\delta =\delta (\varepsilon ,n)$ such that if a measurable event *H* satisfies $\mu (H)<\delta$, then $\mu (H^n)<\varepsilon$, where $H^n:=\{({\omega },x): ({\omega },o)\in H, {{\text {dist}}}_{\omega }(o,x)\le n\}$.

A possible way to view the statement of the lemma is the following: whenever we color a $\delta$ “fraction” of the vertices red, less than an $\varepsilon$ “fraction” of vertices will have a red vertex in distance at most *n*.

#### Proof

It is enough to prove the lemma for $n=1$ because $(H^1)^{n-1}=H^{n}$, and hence $\delta (\varepsilon ,n)=\delta (\delta (\varepsilon ,n-1),1)$. The statement follows by induction.

Fix $\varepsilon$ and let $D=D(\varepsilon )$ be the smallest positive integer that satisfies ${\mathbb {E}}\left( {\mathbf {1}}_{\{\deg o>D\}}\deg o\right) <\varepsilon /4$. We define the following mass transport: let $f(x,y,{\omega })=1$ if $({\omega },x)\in H, ({\omega },y)\notin H, \{x,y\}\in E({\omega })$ (or in the directed case (*x*, *y*) or $(y,x)\in E({\omega })$), and let $f(x,y,{\omega })=0$ otherwise. Then by the Mass Transport Principle

$$\begin{aligned} \mu (H^1\setminus H)&\le \int \sum _{x\in V(G)} f(x,o,{\omega }) d\mu ({\omega },o) = \int \sum _{x\in V(G)} f(o,x,{\omega }) d\mu ({\omega },o)\\&\le {\mathbb {E}}\left( \deg o\cdot {\mathbf {1}}_{\{o\in H\}} \right) \\&\le {\mathbb {E}}\left( D\cdot {\mathbf {1}}_{\{o\in H, \deg o\le D\}} \right) +{\mathbb {E}}\left( \deg o\cdot {\mathbf {1}}_{\{o\in H, \deg o>D\}} \right) \\&\le D\mu (H)+\varepsilon /4, \end{aligned}$$

which is less then $\varepsilon /2$ if $\mu (H)<\frac{\varepsilon }{4D(\varepsilon )}:=\delta (\varepsilon ,1)$. It follows that $\mu (H^1)<\varepsilon$. $\square$

#### Proof of Theorem 6

First we note that by Remark 1 and Proposition 1 it is enough to prove the theorem for non-directed graphs.

Denote the distribution of the limit graph (*G*, *o*) endowed with IID uniform labels *c*(*x*) by $\mu$. Fix *T* and let $\varepsilon _T>0$ be such that if an event *H* satisfies $\mu (H)<\varepsilon _T$, then $\mu (H^{2T+1})<1/T$, as provided by Lemma 4. Let *M*(*T*) be a matching as defined in Lemma 2.

We define the following events: let ${\mathcal {X}}_0:=\{\deg _{M(T)}o=0\}$ and let ${\mathcal {X}}_{i,j}$ be the event that there is an edge $\{o,x\}\in M(T)$, such that *x* has the $i^{th}$ largest label among the neighbors of *o* and *o* has the $j^{th}$ largest label among the neighbors of *x*. Note that the above events are disjoint, $\mu \left( {\mathcal {X}}_0\cup \left( \bigcup _{i,j}{\mathcal {X}}_{i,j}\right) \right) =1$ and if $\{x,y\}\in M(T)$ then $(G,x)\in {\mathcal {X}}_{i,j}$ if and only if $(G,y)\in {\mathcal {X}}_{j,i}$. We can find constants $r=r(T)$ and $d=d(T)$ which satisfy the following: there are disjoint events ${\mathcal {Y}}_{i,j}, i,j\in [d]$ and ${\mathcal {Y}}_0=\left( \cup _{i,j\in [d]}{\mathcal {Y}}_{i,j}\right) ^c$ determined by the labeled neighborhood of radius *r* such that $\mu \left( H\right) <\varepsilon _T$ where $H:=({\mathcal {Y}}_0\bigtriangleup {\mathcal {X}}_0)\cup \left( \bigcup _{i,j\le d}({\mathcal {Y}}_{i,j}\bigtriangleup {\mathcal {X}}_{i,j})\right) \cup \left( \bigcup _{\max \{i,j\}> d}{\mathcal {X}}_{i,j}\right)$, furthermore if $\deg _G o>d$, then $(G,o)\in {\mathcal {Y}}_0$. Denote by ${\mathcal {B}}({\mathcal {Y}}_{i,j})$ the isomorphism types of neighborhoods of radius *r* which determine ${\mathcal {Y}}_{i,j}$.

Now we give all vertices of $G_n$ uniform random [0,1] labels independently and denote the joint distribution of $G_n$ and the labels by $\mu _n$. We define the random matching $M_T(G_n)$ using the labels and the sets ${\mathcal {B}}({\mathcal {Y}}_{i,j})$: let an edge $\{x,y\}$ be in $M_T(G_n)$ iff there is a pair (*i*, *j*) such that $B_{G_n}(x,r)\in {\mathcal {B}}({\mathcal {Y}}_{i,j})$, *y* has the $j^{th}$ largest label among the neighbors of *x*, and $B_{G_n}(y,r)\in {\mathcal {B}}({\mathcal {Y}}_{j,i})$, *x* has the $i^{th}$ largest label among the neighbors of *y*. The edge set $M_T(G_n)$ is a matching, because the events ${\mathcal {B}}({\mathcal {Y}}_{i,j})$ are disjoint. We can define a matching $M_T(G)$ of *G* in the same way. Note, that $M_T(G)$ does not necessarily coincide with *M*(*T*) but it satisfies $|\mu (o\in V(M(T)))-\mu (o\in V(M_T(G)))|<2\varepsilon _T$ by the definition of $M_T(G)$. It follows by Lemma 3 that $\lim _{T\rightarrow \infty }\mu \big (o\in V(M_T(G))\big )=\lim _{T\rightarrow \infty }\mu \big (o\in V(M(T))\big )=m_E(G,o)$.

Denote by ${\mathcal {Q}}_T$ the event that there is an augmenting path for $M_T$ of length less than *T* starting from the root. Let $Q_T(G_n)$ be the random set of vertices *v* of $G_n$ such that $(G_n,v)\in {\mathcal {Q}}_T$ and let $q_T(G_n):=\frac{|Q_T(G_n)|}{|V(G_n)|}$. The event $(G_n,x)\in {\mathcal {Q}}_T$ depends on $B_{G_n}(x,r+2T+1)$ by the definition of $M_T$. Furthermore, in the limiting graph *G*, an augmenting path of length less than *T* can start from *o* only if there is a vertex *x* on that path with $(G,x)\in H$, hence we have ${\mathcal {Q}}_T(G,o)\subseteq H^{2T+1}$. It follows from the convergence $G_n\rightarrow (G,o)$ that

$$\begin{aligned} \lim _{n\rightarrow \infty }{\mathbb {E}}(q_T(G_n))=\lim _{n\rightarrow \infty }\mu _n({\mathcal {Q}}_T(G_n,o))\le \mu (H^{2T+1})<\frac{1}{T}, \end{aligned}$$

hence ${\mathbb {E}}(q_T(G_n))<2/T$ for *n* large enough. We have by Lemma 2.1 of [8], that

7 $$\begin{aligned} \frac{|M_T(G_n)|}{|V(G_n)|}\le m(G_n)\le \frac{T+1}{T}\frac{|M_T(G_n)|}{|V(G_n)|}+q_T(G_n). \end{aligned}$$

Taking expectation in (7) with respect to $\mu _n$, we have for *n* large enough that

$$\begin{aligned} \mu _n\left( o\in V (M_T(G_n))\right) =&\,{\mathbb {E}}\left( \frac{|M_T(G_n)|}{|V(G_n)|}\right) \\ \le {\mathbb {E}}(m(G_n))\le&\frac{T+1}{T}\mu _n\left( o\in V(M_T(G_n))\right) +\frac{2}{T}, \end{aligned}$$

where *o* is a uniform random vertex of $G_n$. Since the event $\{o\in V(M_T(G_n))\}$ depends only on the $(r(T)+1)$-neighborhood of *x*, the convergence of the graph sequence implies $\lim _{n\rightarrow \infty }\mu _n(o\in V(M_T(G_n)))=\mu (o\in V(M_T(G)))$. It follows by letting $T\rightarrow \infty$ that ${\mathbb {E}}(m(G_n))$ converge to $\lim _{T\rightarrow \infty }\mu (o\in M_T(G))=m_E(G,o)$. $\square$

### Almost Sure Convergence of the Directed Matching Ratio

We will examine the network models described in Sect. 1.4. As referred there, each model has a local weak limit, hence Theorem 6 shows that the expected values of the directed matching ratios converge. In this section we will show that almost sure convergence holds as well.

First we note that the local weak convergence of a sequence $G_n$ of random graphs defined on a common probability space does not imply automatically that the sequence converges almost surely in the local weak sense (see Definition 4), as shown by the next example. Let $G_n$ be the path of length $n^2$ or the $n\times n$ square grid, with probability 1/2–1/2. Let the joint distribution of the sequence $G_n$ given by the product measure. Then $G_n$ converges in the local weak sense to the infinite rooted graph *G* which is ${\mathbb {Z}}$ or ${\mathbb {Z}}^2$ with probability 1/2–1/2, but there is almost surely no local weak limit of the deterministic graph sequence given by the product measure. If a sequence $G_n$ of finite random graphs converges almost surely in the local weak sense, then Theorem 6 implies the almost sure convergence of the matching ratio, which will be the case for some of the examined sequences.

#### Remark 5

Skorokhod’s Representation Theorem states that for a weakly convergent sequence $\mu _n\rightarrow \mu$ of probability measures on a complete separable metric space *S* there is a probability space $(\Omega ,{\mathcal {F}},{\mathcal {P}})$ and *S*-valued random variables $X_n$ and *X* with distributions $\mu _n$ and $\mu$, respectively, such that $X_n\rightarrow X$ almost surely.

One could think that Skorokhod’s Theorem could be applied for the graph sequences that we consider, and get the convergence of the matching ratio for almost every sequence, using Theorem 6. This argument does not work for our purpose, because in Skorokhod’s Theorem, the coupling between the finite graphs is coming from the theorem, while in the case of the preferential attachment graphs there is given a joint probability space by construction, that contains them all.

We present two distinct methods to prove the existence of the almost sure limit of the matching ratio of a convergent graph sequence $G_n$. The first one can be applied for the random graph models of Sect. 1.4 that are defined by giving the edges independent orientations. We use this method in Sect. 3.2.1 to prove part (2) of Theorem 2. We show in Lemma 5 that if we give the edges of a converging *deterministic* graph sequence uniform random orientations, then the obtained graph sequence converges almost surely in the local weak sense (see Definition 4) to the same limiting graph with randomly oriented edges. Applying this result to the sequences of Erdős–Rényi random graphs and the random configuration model, which are known to converge almost surely in the non-directed case, the almost sure convergence of the matching ratio follows by Theorem 6.

We apply the approach with the second type of argument to preferential attachment graphs in Sect. 3.2.2. The first method does not apply to this class of graphs, because the orientations of the edges are not independent and we cannot start from an a priory almost sure convergence of the undirected graph sequence. We will show that the matching ratio of $G_n$ is concentrated around its expected value, which together with Theorem 6 on the convergence of the mean of the matching ratio implies the almost sure convergence.

#### Directed Versions of Almost Surely Convergent Graph Sequences—Proof of Part (2) of Theorem 2

In this section we prove Part (2) of Theorem 2. As a consequence, we have that the directed matching ratios of sequences of random regular graphs, graphs given by the random configuration model and Erdős–Rényi random graphs converge almost surely, see Corollary 3, Theorem 2 and Corollary 4, respectively.

First we prove Lemma 5 on the almost sure convergence of a sequence of random directed graphs (see Definition 4) obtained from a convergent deterministic graph sequence by giving independent uniform orientation to the edges. This lemma implies Part (2) of Theorem 2.

The graph sequences examined in this section are known to converge almost surely in the undirected case. It follows by Part (2) of Theorem 2 that their directed matching ratios converge almost surely. By our Proposition 1 and Theorem 2 in [6] on the limit of the matching ratio of convergent graph sequences, one can compute the value of the limit of the directed matching ratio when the limit is a unimodular Galton–Watson tree. In Corollaries 3 and 4 we also present the results given by this argument.

##### Lemma 5

Let $G_n$ be a sequence of deterministic undirected graphs on *n* vertices that converges to the random rooted graph (*G*, *o*) in the local weak sense. Let  be the sequence of random directed graphs obtained from $G_n$ by giving a random uniform orientation to each edge uniformly independently. Then the sequence  converges almost surely in the local weak sense to , which is the random rooted graph obtained from (*G*, *o*) by orienting each edge independently.

##### Proof of Theorem 2

*Part (2)* Consider a sequence  of random directed graphs obtained by giving a uniform random orientations to the edges of a sequence of undirected random graphs $G_n$ that converges almost surely in the local weak sense to the limit graph (*G*, *o*). We have by Lemma 5, that  converges almost surely in the local weak sense to the directed graph . It follows by Theorem 6, that the sequence $m(G_n)$ of the matching ratios converges almost surely to . $\square$

The proof of Lemma 5 essentially follows the proof of Proposition 2.2 in [7]. The main difference is that we had to generalize the proof to graph sequences without a uniform bound on the degrees.

##### Proof of Lemma 5

To handle the case of unbounded degrees, we consider the following neighborhoods of the vertices: for any graph *G* and $v\in V(G)$ denote by $B^-_{G}(v,r)$ the subgraph of *G* obtained from $B_{G}(v,r)$ by removing all edges with both endpoints being at distance *r* from *v*. Then the local weak convergence of the sequence of the finite (directed) random graphs $G_n$ to the rooted random (directed) graph (*G*, *o*) is equivalent with the following: for any *r* and any finite (directed) rooted graph *H* we have $\lim _{n\rightarrow \infty }\mathrm {\mathbb {P}}(B^-_{G_n}(o_n,r)\simeq H)=\mathrm {\mathbb {P}}(B^-_{G}(o,r)\simeq H)$, where $o_n$ is a uniform random vertex of $G_n$.

Fix any positive integer *r* and any finite directed rooted graph . Let *H* be the rooted non-directed graph obtained from  by forgetting the orientations of the edges, and suppose that $\mathrm {\mathbb {P}}(B^-_G(o,r)\simeq H)>0$. Denote by $b(G_n)$ and  the number of vertices *v* of $G_n$ and  such that $B^-_{G_n}(v,r)\simeq H$ and , respectively. We show that  almost surely converges to . Since this holds for any , the lemma follows.

Let *h* be the probability that the graph obtained from *H* by giving each edge a random orientation independently is isomorphic to . Then . We will show that8The statement of the lemma follows from this, because the assumption on the convergence of $G_n$ implies that $\frac{hb(G_n)}{n}$ converges to .

To show (8), we note that if two vertices *x*, *y* in $G_n$ satisfy $B^-_{G_n}(x,r)\simeq B^-_{G_n}(y,r) \simeq H$ and ${{\text {dist}}}_{G_n}(x,y)\ge 2r$, then the orientations of the edges in  are independent. Let *D* be the maximum degree of the graph *H*. We claim that we can define a partition $(R_j^n)_{j=1}^{D^{2r}+1}$ of the set $\{x\in V(G_n): B^-_{G_n}(x,r)\simeq H\}$ such that the distance between any two points of $R_j^n$ is at least 2*r* for every *j* and *n*. Indeed, if ${{\text {dist}}}_{G_n}(x,y)$ is less than 2*r* and $B_{G_n}^-(x,r)\simeq B_{G_n}^-(y,r)\simeq H$, then there is a path of length at most $2r-1$ such that every vertex of that path has distance at most $r-1$ from the set $\{x,y\}$, and hence every vertex in the path has degree at most *D*. It follows, that for any fixed *x*, the number of such paths and hence the number of vertices *y* with ${{\text {dist}}}_{G_n}(x,y)<2r$ is at most $D^{2r}$.

We conclude as in the proof of Proposition 2.2 in [7]. The further part of the proof is essentially the same as the proof of that proposition, but for the sake of completeness we present it here. The graph with vertex set $\{x\in V(G_n): B_{G_n}(x,r)\simeq H\}$ and edge set $\{\{x,y\}:{{\text {dist}}}_{G_n}(x,y)<2r\}$ has maximal degree at most $D^{2r}$, thus there is a coloring of its vertices with $D^{2r}+1$ colors, that gives the partition $(R_j^n)$.

Let $0<\varepsilon <\frac{1}{2}\mathrm {\mathbb {P}}(B^-_G(o,r)\simeq H)$ and $0<\delta$ be arbitrary, and let $R_1^n,\dots ,R_{k(n)}^n$ be the list of the sets $R_j^n$ that satisfy $|R_j^n|\ge \varepsilon ^2|V(G_n)|/(D^{2r}+1)$. Denote by  the number of vertices *v* in $R_j^n$ such that . By the strong law of large numbers9holds for all *n* large enough and $j\le k(n)$ with probability at least $1-\delta$. Using (9), the assumptions on $\varepsilon$ and the size of the sets $R_j^n$ we havefor all large enough *n* with probability at least $1-\delta$. Since $\varepsilon$ and $\delta$ was arbitrary, this implies (8). $\square$

The directed versions of the first three graph models of Sect. 1.4 are given by orienting the edges of the non-directed versions independently. We use the following consequence of Theorem 3.28 in [5] for the almost sure convergence of the directed random configuration model (see Sect. 1.4 for the definition):

##### Theorem 7

([5], Theorem 3.28) If $G_n$ is sequence of random undirected graphs given by the random configuration model with degree distribution $\xi$ satisfying ${\mathbb {E}}(\xi ^p)<\infty$ for some $p>2$, then the sequence $G_n$ converges to $UGW(\xi )$ almost surely in the local weak sense.

A corollary of Part (2) of Theorems 2 and 7 is the almost sure convergence of the sequence of graphs obtained by the random configuration model and the matching ratio of it. The connected component of the root $o'$ of the bipartite representation of  has distribution $UGW(Binom(\xi ,1/2))$, hence the limit of the matching ratio can also be expressed as the expected matching ratio of a non-directed unimodular graph.

##### Corollary 2

(Almost sure convergence of the directed matching ratio of the random configuration model) Let $\xi$ be a non-negative integer valued random variable with ${\mathbb {E}}(\xi ^p)<\infty$ for some $p>2$. Let $G_n$ be a sequence of random directed graphs given by the random configuration model with degree distribution $\xi$, or let $G_n$ be the graph given by the same rule but conditioned to be a simple graph. Then  converge almost surely in the local weak sense to  and  converges almost surely to .

The sequence of random directed *d*-regular graphs is a special case of the random configuration model (with degree distribution $\xi$ being constant *d*). The connected component of the root $o'$ of the bipartite representation ${\mathbb {T}}'_d$ has distribution *UGW*(*Binom*(*d*, 1 / 2)), hence we have the following:

##### Corollary 3

(Almost sure convergence of the directed matching ratios of directed random regular graphs) Let $G_n$ be the sequence of random *d*-regular graphs on *n* vertices with randomly oriented edges. Then the matching ratios converge almost surely to the constant

$$\begin{aligned} \lim _{n\rightarrow \infty }m(G_n)=m_E\big (UGW(Binom(d,1/2))\big ). \end{aligned}$$

##### Remark 6

The constants $m_E\big (UGW(Binom(d,1/2))\big )$ or $m_E\big (UGW(Binom(\xi ,1/2))\big )$ equal $1-\max _{t\in [0,1]}F(t)$ by Theorem 2 in [6], where in the definition of the function *F* we use $\phi (t)=\left( \frac{1+t}{2}\right) ^d$ or $\phi (t)=\phi _\xi \left( \frac{1+t}{2}\right)$, respectively (see the theorem for the definition of *F*).

For directed Erdős–Rényi graphs one can compute the exact value of the almost sure limit of the matching ratio, using the results of [9] or Theorem 2 in [6].

##### Corollary 4

Let  be a sequence of directed Erdős–Rényi graphs with parameter 2*c*. Then almost surely

10 $$\begin{aligned} \lim _{n\rightarrow \infty }m({\mathcal {G}}_{n,2c/n})=1-\frac{t_{c}+e^{-ct_{c}}+ct_{c}e^{-ct_{c}}}{2} \end{aligned}$$

where $t_{c}\in (0,1)$ is the smallest root of $t=e^{-ce^{-ct}}$.

##### Proof

According to Sect. 1.4 and Lemma 5, the sequence of directed Erdős–Rényi random graphs converge almost surely in the local weak sense to , and hence , where *Poi*(2*c*) denotes the Poisson distribution with parameter 2*c*. The connected component of the root in the bipartite representation of  has distribution *UGW*(*Poi*(*c*)), which is the almost sure local weak limit of the non-directed Erdős–Rényi random graphs ${\mathcal {G}}_{n,c/n}$ with parameter *c*. It is known (see [9] or Theorem 2 in [6]), that for this graph sequence $\lim _{n\rightarrow \infty }m({\mathcal {G}}_{n,c/n})$ equals the right hand side of (10) almost surely. By Remark 3 we haveThis proves (10). $\square$

#### Preferential Attachment Graphs—Proof of Part (3) of Theorem 2

In this section we show that the directed matching ratio of a graph sequence given by the preferential attachment rule converges almost surely, see Theorem 8. The orientations of the edges of this class of graphs are given naturally by the recursive definition, and differ significantly from the independent random orientation. Thus we cannot apply the results of Sect. 3.2.1. This sequence also does not satisfy the assumption of [10] that the distributions of the in- and out-degrees are the same (which was assumed to simplify the calculations made there), hence the value of the limit of the matching ratio for this class was not examined in that paper. However, the almost sure convergence of the directed matching ratios holds for this class of graph sequences as well, as we show in the next theorem.

##### Theorem 8

(Almost sure convergence of the matching ratio of preferential attachment graphs) Let $G_n$ be a random graph sequence obtained by the preferential attachment rule. Then $m(G_n)$ converges almost surely, namely $\lim _{n\rightarrow \infty }m(G_n)=\lim _{n\rightarrow \infty }{\mathbb {E}}(m(G_n))$ almost surely.

We will prove the concentration of the matching ratios around their expected value, which together with the results of [3] on the local weak convergence of $G_n$ and Theorem 6 on the convergence of the mean of the matching ratio implies the statement.

##### Remark 7

It follows from the concentration shown in the proof, that the almost sure local weak convergence holds for any joint distribution of the graphs $G_n$.

##### Proof of Theorem 8

Fix *n* and denote by $G_n[k]$ the subgraph of $G_n$ spanned by the vertices $\{1,\dots ,k\}$. Let

11 $$\begin{aligned} X_k:={\mathbb {E}}\left( |M_{max}(G_n)|\,\Big |\,G_n[k]\right) -{\mathbb {E}}\left( |M_{max}(G_n)|\,\Big |\,G_n[k-1]\right) . \end{aligned}$$

We will show that $|X_k|\le 2r$ almost surely for all $k\in [n]$. Since $Y_k:={\mathbb {E}}\left( |M_{max}(G_n)|\Big |G_n[k]\right)$ is a martingale, we can apply the Azuma–Hoeffding inequality (Theorem 5) to the random variables $X_k$. It follows that for any $c>0$ we have

$$\begin{aligned} \mathrm {\mathbb {P}}\left( |m(G_n)-{\mathbb {E}}(m(G_n))|>c \right)&=\mathrm {\mathbb {P}}\left( |X_1+\dots +X_n|>cn \right) \\&\le 2\exp \left\{ -\frac{(cn)^2}{2\sum _{k=1}^n \Vert X_k\Vert _\infty ^2} \right\} \\&\le 2\exp \left\{ -\frac{c^2n^2}{8nr^2} \right\} . \end{aligned}$$

Since $\lim _{k\rightarrow \infty }{\mathbb {E}}(m(G_k))$ exists by Theorem 6, for *n* large enough to satisfy $|{\mathbb {E}}(m(G_n))-\lim _{k\rightarrow \infty }{\mathbb {E}}(m(G_k))|<c/2$ we have

$$\begin{aligned} \mathrm {\mathbb {P}}\left( |m(G_n)-\lim _{k\rightarrow \infty }{\mathbb {E}}(m(G_k))|>c \right)&\le \mathrm {\mathbb {P}}\left( |m(G_n)-{\mathbb {E}}(m(G_n))|>\frac{c}{2} \right) \\&\le 2\exp \left\{ -\frac{c^2n}{32r^2} \right\} . \end{aligned}$$

It follows that these probabilities are summable in *n* for every $c>0$, which implies the almost sure convergence of $m(G_n)$ by the Borel–Cantelli lemma.

What remains to show is that for any fixed pair of directed graphs *F* and $F'$ on the vertex set [*k*] with $F[k-1]=F'[k-1]$, the inequality

12 $$\begin{aligned} \bigg |{\mathbb {E}}\Big (|M_{max}(G_n)|\,\Big |\, G_n[k]=F \Big )-{\mathbb {E}}\Big (|M_{max}(G_n)|\,\Big |\, G_n[k]=F' \Big )\bigg |\le 2r \end{aligned}$$

holds. This implies $|X_k|\le 2r$.

Fix *F* and $F'$ as above. For any possible configuration of $G_n$, denote by

$$\begin{aligned} h(G_n):=\left\{ (\ell ,j)\in E(G_n): \ell >k, (k,j)\notin E(F)\cup E(F')\right\} \end{aligned}$$

the subset of the edges of $G_n$ with tails in $\{k+1,\dots ,n\}$ that do not have a common head with the edges in the graphs *F* or $F'$ with tail *k*. The proof of inequality (12) is based on two observations: first, by the definition of the preferential attachment graph, the distribution of $h(G_n)$ conditioned on $\{G_n[k]=F\}$ is the same as conditioned on $\{G_n[k]=F'\}$ (note the symmetry in *F* and $F'$ in the definition of $h(G_n)$). Second, for any configuration of $G_n$ with $G_n[k]=F$, the size of the maximal matching changes by at most 2*r* if we fix $h(G_n)$, set $G_n[k]:=F'$ and vary arbitrary the heads of the edges with tails in $\{k+1,\dots ,n\}$ that are not in $h(G_n)$. This follows from Lemma 1 by the following argument. For any fixed *H* we obtain any graph in the set $\{G_n:G_n[k]=F,h(G_n)=H\}$ by adding new edges with heads in the set $\{j: (k,j)\in E(F)\cup E(F')\}$ of size at most 2*r* to the graph $G_H$ with $V(G_H):=[n]$ and $E(G_H):=E(F[k-1])\cup H$. It follows from Lemma 1 that

13 $$\begin{aligned} |M_{max}(G_H)|&\le {\mathbb {E}}\Big (|M_{max}(G_n)|\,\Big |\,G_n[k]=F,h(G_n)=H\Big ) \nonumber \\&\le |M_{max}(G_H)|+2r, \end{aligned}$$

and the same holds with $F'$ in the place of *F*. This proves the second observation.

Using the first observation and (13) the left hand side of (12) can be estimated from above by

$$\begin{aligned}&\sum _{H} \bigg | {\mathbb {E}}\left( |M_{max}(G_n)|\Big |G_n[k]=F,h(G_n)=H\right) \mathrm {\mathbb {P}}\left( h(G_n)=H\Big |G_n[k]=F\right) \\&\quad \qquad - {\mathbb {E}}\left( |M_{max}(G_n)|\Big |G_n[k]=F',h(G_n)=H\right) \mathrm {\mathbb {P}}\left( h(G_n)=H\Big |G_n[k]=F'\right) \bigg | \\&\qquad \le \sum _{H} \mathrm {\mathbb {P}}\left( h(G_n)=H\,\Big |\,G_n[k-1]=F[k-1]\right) \\&\quad \qquad \cdot \bigg | {\mathbb {E}}\left( |M_{max}(G_n)|\,\Big |\,G_n[k]=F,h(G_n)=H\right) \\&\quad \qquad -{\mathbb {E}}\left( |M_{max}(G_n)|\,\Big |\,G_n[k]=F',h(G_n)=H\right) \bigg | \\&\quad \le \sum _{H} \mathrm {\mathbb {P}}\left( h(G_n)=H\,\Big |\,G_n[k-1]=F[k-1]\right) \cdot 2r \\&\quad =2r \end{aligned}$$

$$\square $$

## References

[1] Barabási A-L, Albert R (1999). Emergence of scaling in random networks. Science.

[2] Benjamini, I., Schramm, O.: Recurrence of distributional limits of finite planar graphs. Electron. J. Probab. **6** (2001)

[3] Berger N, Borgs C, Chayes JT, Saberi A (2014). Asymptotic behavior and distributional limits of preferential attachment graphs. Ann. Probab..

[4] Bollobás B, Riordan O (2004). The diameter of a scale-free random graph. Combinatorica.

[5] Bordenave, Ch.: Lecture notes on random graphs and probabilistic combinatorial optimalization (2016). https://www.math.univ-toulouse.fr/~bordenave/coursRG.pdf. Accessed 15 Sept 2018

[6] Bordenave Ch, Lelarge M, Salez J (2013). Matchings on infinite graphs. Probab. Theory Relat. Fields.

[7] Elek G (2010). Parameter testing in bounded degree graphs of subexponential growth. Random Struct. Algorithm..

[8] Elek G, Lippner G (2010). Borel oracles. An analytical approach to constant-time algorithms. Proc. Am. Math. Soc..

[9] Karp, R., Sipser, M.: Maximum matchings in sparse random graphs. In: IEEE Proceedings of the Twenty Second Annual Symposium on Foundations of Computer Science, pp. 364–375 (1981)

[10] Liu Y-Y, Slotine J-J, Barabási A-L (2011). Controllability of complex networks. Nature.

[11] Liu Y-Y, Csóka E, Zhou H, Pósfai M (2012). Core percolation on complex networks. Phys. Rev. Lett..

[12] Lyons R, Nazarov F (2011). Perfect matchings as IID factors on non-amenable groups. Eur. J. Comb..

[13] Lyons R, Peres Y (2016). Probability on Trees and Networks.

